# Flavonones from *Penthorum chinense* Ameliorate Hepatic Steatosis by Activating the SIRT1/AMPK Pathway in HepG2 Cells

**DOI:** 10.3390/ijms19092555

**Published:** 2018-08-28

**Authors:** Wei-Wei Guo, Xing Wang, Xiao-Qing Chen, Yin-Ying Ba, Nan Zhang, Rong-Rong Xu, Wen-Wen Zhao, Xia Wu

**Affiliations:** Beijing Key Lab of TCM Collateral Disease Theory Research, School of Traditional Chinese Medicine, Capital Medical University, 10 Youanmen, Xitoutiao, Beijing 100069, China; gww1023@163.com (W.-W.G.); kingstar1016@sina.com (X.W.); cxqcpu@163.com (X.-Q.C.); byy3333@sina.com (Y.-Y.B.); nan623@126.com (N.Z.); xrr9515@163.com (R.-R.X.); wenwenzhao1994@163.com (W.-W.Z.)

**Keywords:** *Penthorum chinense* Pursh, NAFLD, hepatic steatosis, flavonoids, SIRT1, AMPK

## Abstract

Pinocembrin-7-*O-β*-d-glucoside (PCBG), pinocembrin (PCB), and 5-methoxy-pinocembrin-7-*O-β-*d-glucoside (MPG) are three flavonones isolated from *Penthorum chinense* Pursh (*P. chinense*). The effects of the three flavonones on hepatic steatosis and their molecular mechanisms in HepG2 cells were investigated in this study for the first time. A model of hepatic steatosis in HepG2 cells was induced by free fatty acid (FFA), and co-treated with the three flavonones as mentioned. Intracellular lipid droplets were detected by Oil Red O staining. PCB, PCBG, and MPG suppressed oxidative stress by decreasing malondialdehyde (MDA) levels and increasing superoxide dismutase (SOD) and glutathione peroxidase (GSH-Px) activities. The levels of aspartate aminotransferase (AST) and alanine aminotransferase (ALT) were ameliorated. Moreover, these flavonones enhanced the phosphorylation of AMP-activated protein kinase (AMPK) and the expression of silent mating type information regulation 2 homolog 1 (SIRT1) and peroxisome proliferator-activated receptor α (PPARα), and reduced the expression of sterol regulatory element binding protein-1c (SREBP1c) and the downstream targets fatty acid synthase (FAS), acetyl-CoA carboxylase (ACC), and stearoyl-CoA desaturase 1 (SCD1). Molecular docking was used to predict the interaction and combination patterns between the three flavonones and the enzymes above. The results revealed that the SIRT1/AMPK pathway is involved in the functions of the three flavonones, and the most effective flavonone against hepatic steatosis might be PCBG, followed by MPG and PCB. Therefore, the three flavonones from *P. chinense* were found to exert preventive effects against hepatic steatosis by regulating the SIRT1/AMPK pathway.

## 1. Introdution

Non-alcoholic fatty liver disease (NAFLD) is now the most common chronic liver disease in the world [1]. Currently, the prevalence of NAFLD in Asia is around 25%, similar to that in many Western countries [2]. NAFLD is characterized as a metabolic syndrome, which is associated with insulin resistance, obesity, and dyslipidemia [3]. NAFLD ranges from simple steatosis, steatohepatitis, and fibrosis to cirrhosis, and the prevalence of non-alcoholic steatohepatitis (NASH), developed from NAFLD, is estimated at 3–5% in the general population [4,5]. People with NASH have a much higher risk of liver fibrosis, cirrhosis, and even hepatocellular carcinoma [6,7].

Several studies concluded that weight loss, dietary interventions, and physical activity could potentially ameliorate biochemical, histological, and structural abnormalities of NAFLD, whereas drugs, such as statins, vitamin E, glitazones, and metformin are used to reduce the amelioration [5,8,9]. The pathogenesis of NAFLD is complex and not completely understood, although increased visceral adiposity and insulin resistance with increased free fatty acid (FFA) release are confirmed to play an important role in the development of liver steatosis [10]. AMP-activated protein kinase (AMPK) and silent mating type information regulation 2 homolog 1 (SIRT1) are the key enzymes responsible for longevity and energy homeostasis by regulating glucose and lipid metabolism in a finely tuned network [11,12,13]. AMPK stimulation during fatty acid metabolism is presented as AMPK phosphorylation, and it is known as a critical regulator for sterol regulatory element binding protein-1 (SREBP1) activation and lipogenesis [14,15]. Sterol regulatory element binding protein-1c (SREBP1c) regulates gene expression related to glucose metabolism, fatty acid, and lipid production, and its activity is regulated by insulin [16]. Moreover, SREBP1c can up-regulate the transcription of fatty acid synthase (FAS), stearoyl-CoA desaturase 1 (SCD1), and acetyl-CoA carboxylase (ACC) [17,18], which primarily catalyzes the synthesis of long-chain fatty acids from acetyl-CoA and malonyl-CoA [19,20]. 

*Penthorum chinense* Pursh (*P. chinense*), a Chinese medicine in the family of Saxifragaceage, is used as folk medicine and a functional drink with antioxidant, anti-complement, and liver-protecting effects [21,22]. A previous study showed that *P. chinense* extract has effects on NAFLD treatment [23]. Gansu granules, made from *P. chinense* extract, are widely used in the clinic for various ailments of the liver, such as chronic hepatitis B and NAFLD [24,25]. Pinocembrin-7-*O-β*-d-glucoside (PCBG), pinocembrin (PCB), and 5-methoxy-pinocembrin-7-*O-β-*d-glucoside (MPG) are three flavonones with similar nuclear structures isolated from the extract of *P. chinense*. PCBG and PCB were reported to possess hepatoprotective, antioxidant, anti-inflammatory, and anti-hepatocarcinoma activities [26,27]. Moreover, our previous studies identified MPG as a new flavonone [28], and PCBG can be degraded into PCB rapidly not only in pharmacokinetic studies in vivo, but also in biotransformation in vitro [29]. However, there is no report on NAFLD treatment using these three ingredients.

To investigate the resistant effects of PCBG, PCB, and MPG on NAFLD and their possible therapeutic mechanism, targets on the SIRT1/AMPK/SREBP1c pathway related to lipid metabolism were evaluated in a nonalcoholic injured HepG2 cell model induced by FFA in the present study. Moreover, molecular docking of the binding of the three flavonones to several targets in the lipid metabolic pathway was performed to determine the ligand–protein binding interaction in silico. We hypothesized that the flavonones in *P. chinense* produced their hepatoprotective action via the regulation of lipid generation and metabolism, and in vivo research will be performed in our group.

## 2. Results

### 2.1. FFA-Induced Cytotoxicity and Concentration Screening of PCB, PCBG, and MPG

**Figure 1:**
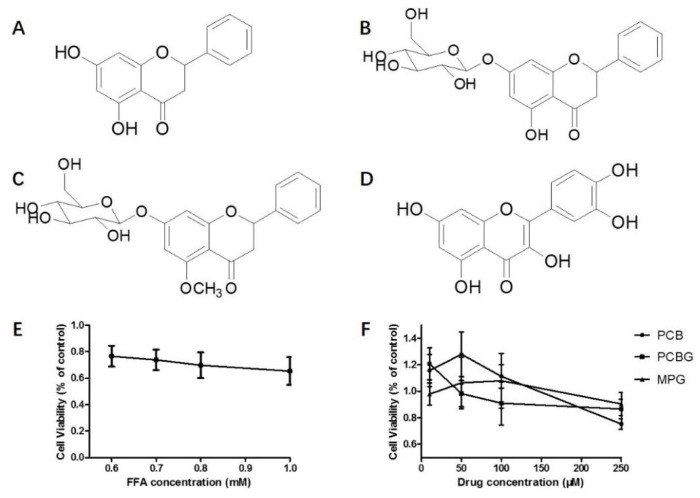
Structures of pinocembrin (PCB; **A**), pinocembrin-7-*O-β*-d-glucoside (PCBG; **B**), fcg 5-methoxy-pinocembrin-7-*O-β-*d-glucoside (MPG; **C**), and reference quercetin (QCT; **D**). The cytotoxicity of free fatty acid (FFA; **E**) and the three compounds (**F**) toward HepG2 cells. The experiments were performed at least three times independently, and the results are displayed as mean ± SD.

The effects of FFA and drugs on the viability of HepG2 cells were assessed. As shown in Figure 1E,F, 0.8 mM FFA reduced cell viability to approximately 70%, and concentrations of 10, 50, and 100 μM PCB and MPG were not cytotoxic to HepG2 cells. A concentration range below 10 μM of PCBG was not cytotoxic. Thus, FFA (0.8 mM) and co-treatment with PCB, MPG (1, 10, and 100 μM), and PCBG (0.1, 1, and 10 μM) were used to evaluate drug effects in HepG2 cells in this study.

### 2.2. PCB, PCBG, and MPG Inhibited Intracellular Lipid Accumulation in HepG2 Cells

**Figure 2:**
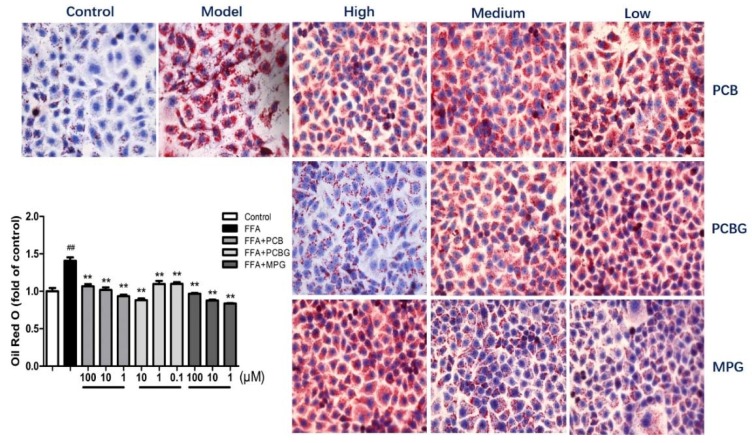
Qualitive and quantitative measurements of hepatic lipid accumulation in the HepG2 cells as observed by Oil Red O staining (original magnification 400×). Data represent the mean ± SD of five independent experiments. ^##^ *p* < 0.01 versus control; ** *p* < 0.01 versus FFA group.

To verify the inhibition of FFA-induced lipid accumulation by PCB, PCBG, and MPG, the cells were stained with Oil Red O, then observed under the microscope and quantified by measuring the absorbance at 510 nm. Oil Red O staining showed more lipid droplets in HepG2 cells after FFA treatment compared to the control group (Figure 2). The lipids accumulated in the presence of FFA (0.8 mM); however, co-treatment of FFA with PCB, PCBG, and MPG significantly declined the number of lipid droplets in a dose-dependent manner (*p* < 0.01). Quantitative measurements also showed that treatment with PCB, PCBG, and MPG alleviated the FFA-induced accumulation of triglycerides.

### 2.3. PCB, PCBG, and MPG Weakened Lipid Levels and Up-Regulated Antioxidant Enzymes

**Figure 3:**
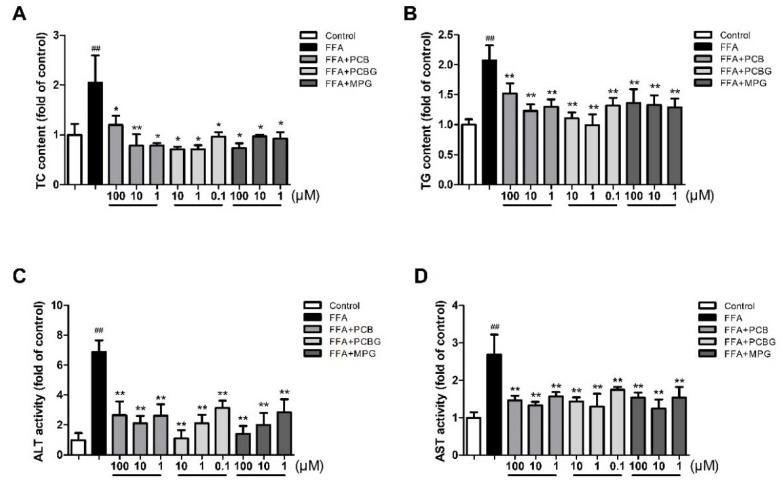
The effects of PCB, PCBG, and MPG on total cholesterol (TC; **A**), triglyceride (TG; **B**), alanine aminotransferase (ALT; **C**), and aspartate aminotransferase (AST; **D**) levels in HepG2 cells. The experiments were performed at least three times independently, and the results are displayed as mean ± SD. ^##^ *p* < 0.01 versus control; * *p* < 0.05 versus FFA group; ** *p* < 0.01 versus FFA group.

To evaluate the effect of PCB, PCBG, and MPG on liver function, an enzymatic method was used to evaluate liver function. FFA treatment caused severe liver function injury in HepG2 cells, as indicated by the increase in alanine aminotransferase (ALT) and aspartate aminotransferase (AST) activities (*p* < 0.01). Treatment with PCB, PCBG, and MPG at three doses significantly blocked the increase in ALT and AST activities in the presence of FFA. Analysis of hepatic total cholesterol (TC) and triglyceride (TG) contents confirmed hepatic steatosis by FFA. Intracellular TG and TC levels were increased by FFA treatment, but this effect was blocked by PCB, PCBG, and MPG (Figure 3). 

**Figure 4:**
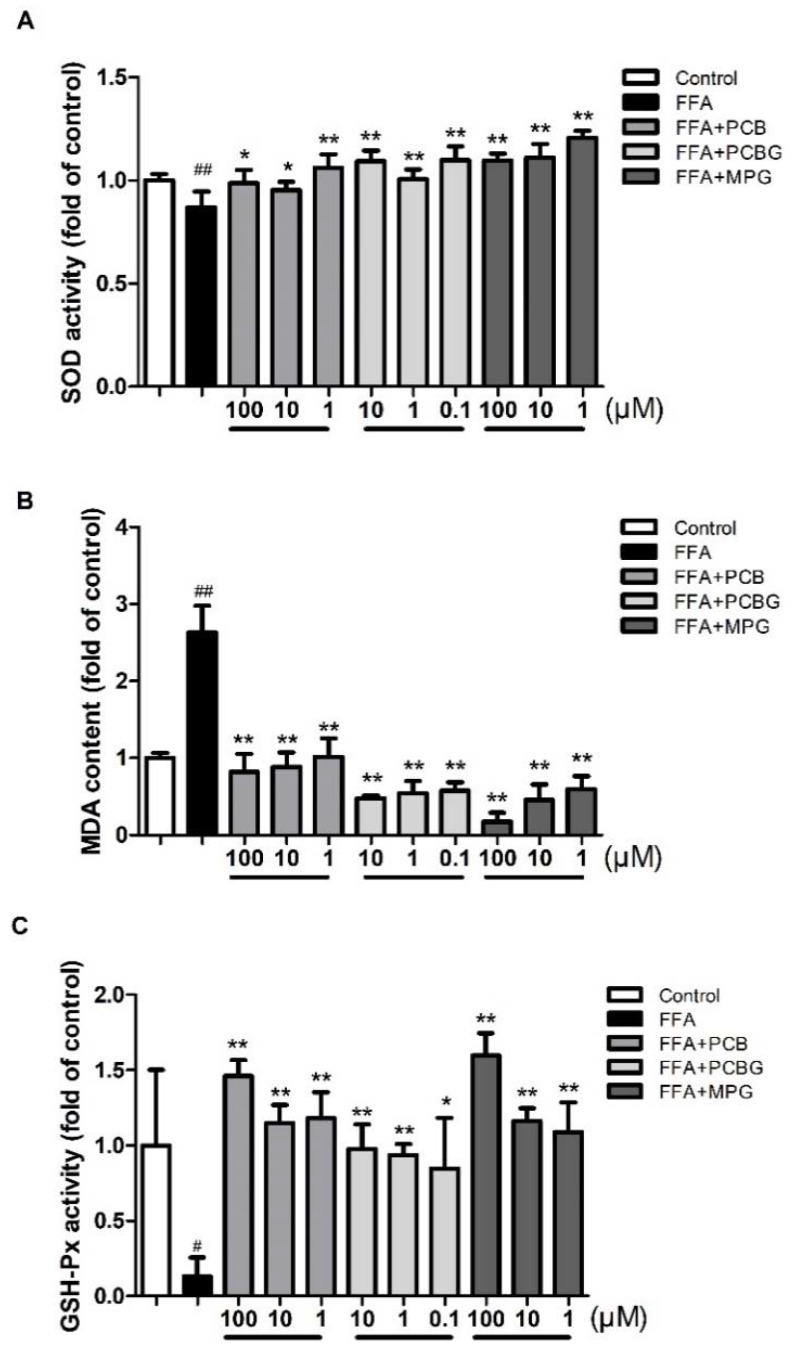
The effects of PCB, PCBG, and MPG on superoxide dismutase (SOD; **A**), malondialdehyde (MDA; **B**), and glutathione peroxidase (GSH-Px; **C**) levels in HepG2 cells. The experiments were performed at least three times independently and the results are displayed as mean ± SD. ^#^ *p* < 0.05 versus control; ^##^ *p* < 0.01 versus control; * *p* < 0.05 versus FFA group; ** *p* < 0.01 versus FFA group.

The effects of each treatment on the levels of oxidative stress are shown in Figure 4. Compared to the control group, the activities of superoxide dismutase (SOD) and glutathione peroxidase (GSH-Px) in FFA-treated HepG2 cells were significantly reduced, while malondialdehyde (MDA) levels were increased, indicating that antioxidant activity was reduced, but lipid peroxidation was increased. Furthermore, PCB, PCBG, and MPG treatments significantly enhanced SOD (*p* < 0.05) and GSH-Px (*p* < 0.05) activities, and decreased MDA (*p* < 0.01) levels when compared with FFA-treated cells. The beneficial role of MPG on the level of oxidative stress was stronger than PCB and PCBG, and presented a dose-dependent manner.

### 2.4. Effects of PCB, PCBG, and MPG on the Expression of Factors Associated with Hepatic Lipid Accumulation

**Figure 5:**
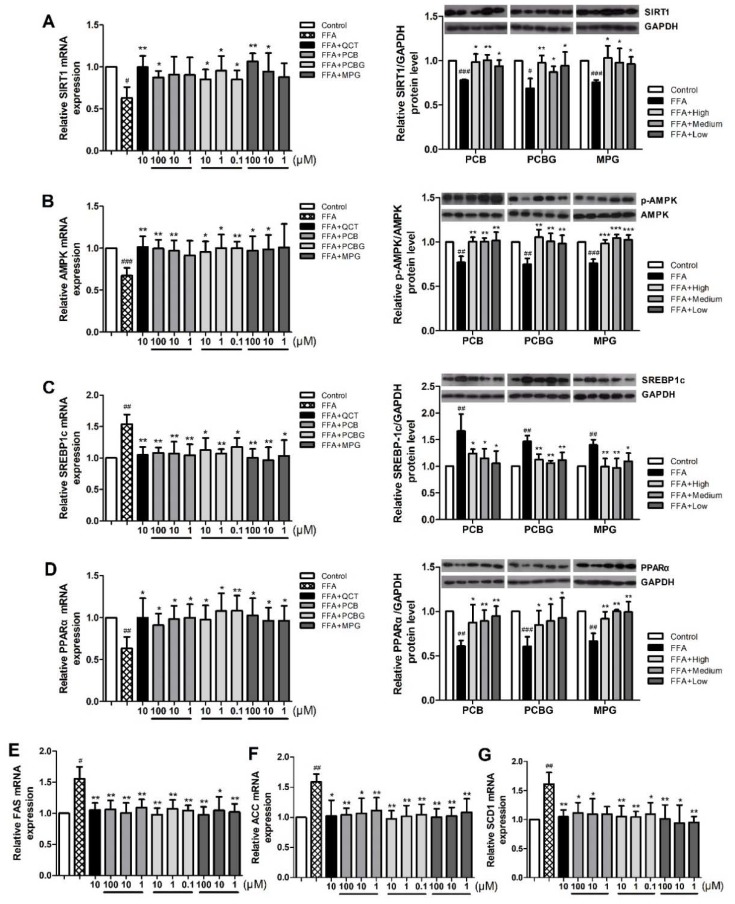
The effects of PCB, PCBG, and MPG on hepatic steatosis depends on the silent mating type information regulation 2 homolog 1/AMP-activated protein kinase (SIRT1/AMPK) pathway. (**A**) Effect on mRNA and protein expressions of SIRT1. (**B**) Effect on AMPK mRNA expression and phosphorylated (p)-AMPK/AMPK protein levels. (**C**) Effect on mRNA and protein expressions of sterol regulatory element binding protein-1c (SREBP1c). (**D**) Effect on mRNA and protein expressions of peroxisome proliferator-activated receptor α (PPARα). (**E**–**G**) Effects on fatty acid synthase (FAS), acetyl-CoA carboxylase (ACC), and stearoyl-CoA desaturase 1 (SCD1) protein levels. The experiments were performed at least four times independently and the results are displayed as mean ± SD. ^#^ *p* < 0.05 versus control; ^##^ *p* < 0.01 versus control; ^###^ *p* < 0.001 versus control; * *p* < 0.05 versus FFA group; ** *p* < 0.01 versus FFA group; *** *p* < 0.001 versus FFA group.

To determine the alternation of de novo lipogenesis in response to FFA and the three flavonones, the mRNA expressions of SREBP1c and its target enzymes, FAS, ACC, and SCD1 were examined using qRT-PCR and compared with the mRNA expression of quercetin (QCT). As shown in Figure 5, FFA enhanced their mRNA expressions in HepG2 cells, which were attenuated by PCB, PCBG, and MPG treatment, particularly by high and medium concentrations of PCBG and MPG. The protein expressions of peroxisome proliferator-activated receptor α (PPARα), SREBP1c, FAS, ACC, and SCD1 were assessed using Western blot. Compared to the control group, FFA treatment enhanced the protein expressions of SREBP1c, FAS, ACC, and SCD1 (Figure 5E–G). Moreover, FFA induced a remarkable decrease in PPARα protein expression. When co-treated with PCB, PCBG, and MPG, the FFA-induced alternations in the proteins for de novo lipogenesis were ameliorated significantly. More specifically, PCB, PCBG, and MPG at medium concentration down-regulated SREBP1c protein levels by 30.64%, 27.67% and 30.81% (Figure 5C), respectively, and recovered PPARα protein expression by 46.65%, 47.12% and 50.04% (Figure 5D), respectively. The results suggest that PCB, PCBG, and MPG attenuated HepG2 cells induced by FFA via the de novo lipogenesis pathway.

### 2.5. Effects of PCB, PCBG, and MPG on AMPK and SIRT1 Activities in HepG2 Cells

Activated AMPK reduces lipogenesis and lipid accumulation by suppressing SREBP1c cleavage and nuclear translocation [30,31]. To further evaluate the mechanism for the roles of PCB, PCBG, and MPG in relieving fatty liver, we assessed the phosphorylation of AMPK and SIRT1 using qRT-PCR and Western blot. The expression of phosphorylated (p)-AMPK decreased significantly in the FFA group compared with the control group, and this effect was blocked in the presence of PCB and MPG at 100 and 10 μM, and at all three concentrations of PCBG. SIRT1 and AMPK are two key enzymes responsible for longevity and energy homeostasis. The expression and deacetylation activities of SIRT1 are enhanced by the increase in oxidized nicotinamide adenine dinucleotide (NAD^+^) levels or the ratio of NAD^+^ to reduced nicotinamide adenine dinucleotide (NADH), which was suggested by the activation of AMPK to some extent [32]. Therefore, we studied the mRNA and protein expressions of SIRT1, and found that FFA treatment reduced SIRT1 expression, which was recovered by PCB and MPG at 100 and 10 μM, and was significantly up-regulated by PCBG.

### 2.6. Docking Studies

**Figure 6:**
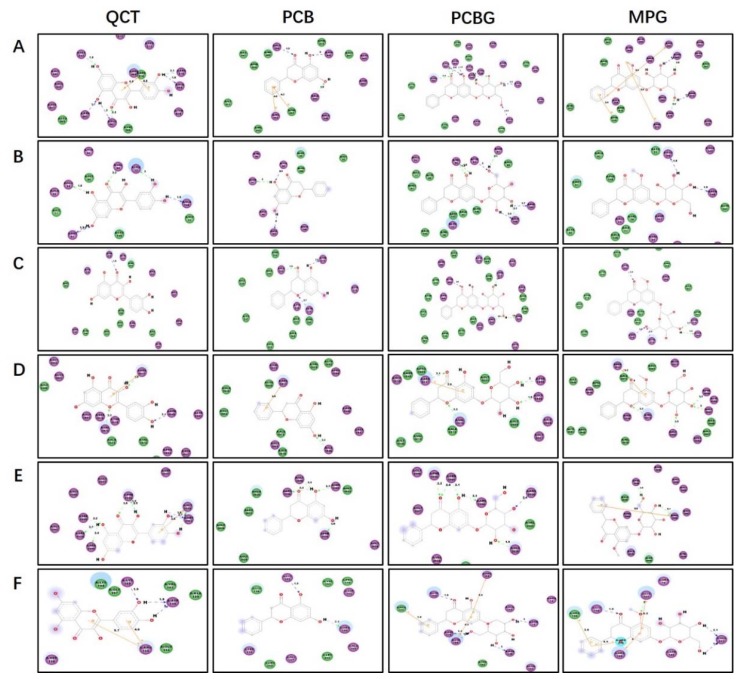
Molecular interactions of PCB, PCBG, MPG, and QCT binding with (**A**) SIRT1, (**B**) AMPK, (**C**) PPARα, (**D**) FAS, (**E**) ACC1, and (**F**) SCD1. Sharper images are in Supplementary Figure S1.

Molecular docking studies were performed to investigate the interactions between targets including SIRT1, AMPK, PPARα, FAS, ACC1, and SCD1 and ligands including PCB, PCBG, MPG and the reference compound, QCT. The docking score and binding mode were evaluated with docking studies (Table 1), and images of the compounds with amino acids involved in binding poses are shown in Figure 6. PCBG showed a higher docking score for binding with AMPK, FAS, and ACC1 than QCT. MPG showed better AMPK, PPARα, and FAS binding action than QCT. However, PCB showed weak binding with the selected proteins.

## 3. Discussion

NAFLD, a metabolic syndrome, is a major health problem. The reported prevalence of NAFLD is up to 20% in the general population worldwide [5]. NAFLD is characterized by increased fatty-acid uptake, de novo lipogenesis, reduced fatty-acid oxidation, and very-low-density lipoprotein (VLDL) secretion. Flavonoids were reported to exert multiple benefits on the disorders associated with NAFLD [33]. *P. chinense* was reported to possess antioxidant and hepatoprotective activities [21,22]. Previous studies revealed that *P. chinense* is rich in flavonoids [34] and that these flavonoids play roles in protecting the liver [26,27]. The three selected compounds had the same structure in the nucleus. MPG is a new flavonone, while PCBG is the highest-level flavonone in *P. chinense*. In this study, the effects and mechanisms of PCB, PCBG, and MPG on a nonalcoholic injured HepG2 cell model were investigated for the first time.

FFA-induced lipid accumulation in hepatocytes is a commonly used model to study hepatic steatosis [35,36]. These fatty acids may be converted into other lipid species, such as glycerolipids, glycerophospholipids, and sterols. Fatty-acid oxidation damages the mitochondrial function of cells [37]. The HepG2 cells were co-treated with FFA and three doses of PCB, PCBG, and MPG, and the doses were decided upon screening cell cytotoxicity. The results of Oil Red O staining showed that PCB, PCBG, and MPG declined the number of lipid droplets in a dose-dependent manner. FFA treatment increased the concentrations of TC and TG, as well as the levels of AST and ALT, which reflected the extent of hepatocyte damage and hepatic steatosis [38]. Flavonoids significantly reduced FFA-induced changes in TC, TG, AST, and ALT, indicating their protective effects on liver. These results suggest that PCB, PCBG, and MPG inhibited FFA-induced lipid accumulation. PCBG inhibited the activity of ALT in a dose-dependent manner (high vs. medium, *p* < 0.05; medium vs. low, *p* < 0.01; high vs. low, *p* < 0.001).

Free radicals may lead to cell damage not only by lipid peroxidation, but also through decomposition products of lipid hydroperoxides, while flavonoids show good antioxidative effects [39]. Therefore, parameters including MDA, SOD, and GSH-Px were measured to evaluate the oxidative stress. MDA, as the product of lipid peroxidation, indirectly reflects the severity of attack by free radicals. SOD is in charge of catalytic dismutation of free radicals and reducing superoxide levels, which reflects the ability to scavenge free radicals [40]. GSH-Px specifically catalyzes the decomposition of hydrogen peroxide to protect the integrity of cell membrane structure and function. The increased concentration of SOD levels and the decreased levels of MDA and GSH-Px in flavonoid-treated cells proved their protective effect. Furthermore, the effect of MPG occurred in a dose-dependent manner with significant differences between high and low levels of MPG (*p* < 0.01).

**Figure 7:**
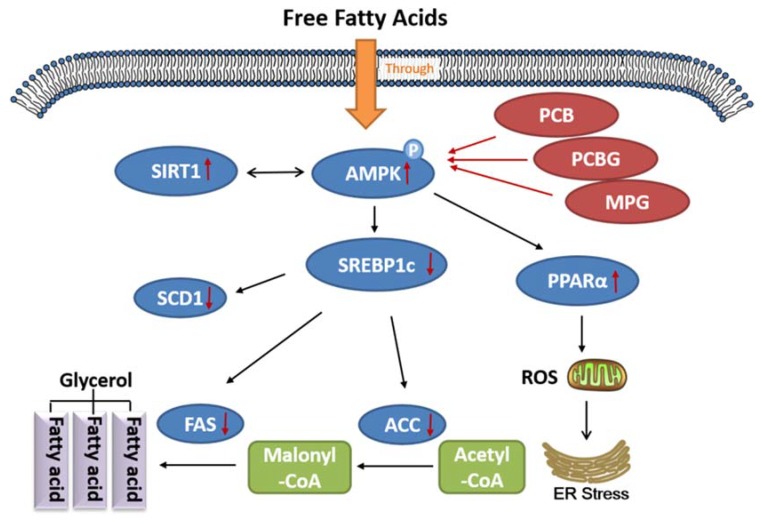
Schematic diagram presenting pathways via which PCB, PCBG, and MPG ameliorate hepatic steatosis by activating the SIRT1/AMPK pathway. Red arrows upward present increased protein expression; Red arrows downward present decreased protein expression.

The SIRT1/AMPK-SREBP1c pathway is key in regulating lipid metabolism [41]. Both SIRT1 and AMPK are known to regulate each other and share many common target molecules, and the interaction between SIRT1 and AMPK could be reciprocal [42]. AMPK is a protein that regulates mitochondrial biogenesis, fatty-acid synthesis, and oxidative metabolism in response to energy deprivation. SIRT1 was shown to be the primary mediator for regulating the expression levels of mitochondrial metabolism genes and lipid metabolism, as well as the consumption of O_2_. Both AMPK and SIRT1 act in concert with the master regulator of mitochondrial biogenesis to regulate energy homeostasis in response to environmental and nutritional stimuli [11,43,44]. AMPK inhibits the rate-limiting enzyme, SREBP1c, in lipogenesis, which leads to decreased lipid deposition [45,46]. SREBP1c is located in the upstream promoter region up-regulating the transcriptions of FAS, ACC, and SCD1, which directly catalyze lipogenesis [18,47]. Moreover, PPARα, known as the ligand-activated nuclear receptor, regulates lipid homeostasis genes [48]. We detected the expression of these genes after treatment with PCB, PCBG, MPG, and the reference compound, QCT, which was reported to exert a preventive effect against hepatic steatosis probably through SIRT1/AMPK and PPARα pathways [49,50]. Our data showed that PCBG (0.1, 1, and 10 μM) and MPG (10 and 100 μM) significantly enhanced SIRT1 and AMPK gene expression, but only 100 μM PCB showed significant improvement on SIRT1 expression, which led to a significant reduction in SREBP1c levels, followed by reduced FAS, ACC, and SCD1 expressions. From the results, it was observed that PCBG (0.1, 1, and 10 μM) and MPG (10 and 100 μM) had appreciably similar hypolipidemic effects to reference QCT (10 μM), while PCB (100 μM) had a visible effect on hepatic steatosis, but weaker than that of QCT (10 μM). A series of recent studies showed that the effects of the active components in traditional Chinese herbs on NAFLD are associated with activating the AMPK signaling pathway, improving insulin resistance, modulating the activity and expression of peroxisome proliferator-activated receptor γ, antioxidant and anti-inflammatory activities, and regulating intestinal flora [51]. These three flavonones might have effects on different potential therapeutic targets. The docking results suggested that binding with polyhydroxy ligands might get higher scores by forming hydrogen-bond interactions with side chains. The C3 carbonyl group and C5 hydroxyl or methoxy group of flavonoids acted as the key hydrogen-bond acceptors by interacting with the amino-acid residues in the active-site region of proteins. The docking score of the compounds was in the order of PCBG, QCT, MPG, and PCB. Finally, this is of great significance to study the pharmacodynamic basis of *P. chinense* and mechanisms on ameliorating hepatic steatosis which was not reported before now. The potency against hepatic steatosis on targets of these flavonoids via molecular docking was consistent with the pharmacophoric features from the cell model (Figure 7).

## 4. Materials and Methods

### 4.1. Cell Culture

HepG2 cells were obtained from the American Type Culture Collection (ATCC; Manassas, VA, USA). Cells were cultured in Dulbecco’s modified Eagle’s medium (DMEM) (Gibco Invitrogen Corporation, Carlsbad, CA, USA) supplemented with 10% fetal bovine serum (FBS) (Gibco Invitrogen Corporation, Carlsbad, CA, USA) in an incubator with 5% CO_2_ at 37 °C. The cells were seeded at 70% confluence in six-well plates and were grown in serum-free DMEM containing 0.5% bovine serum albumin (BSA) (Sigma-Aldrich Co., St. Louis, MO, USA) for 12 h before treatment. Cells of the control group were incubated in DMEM containing 0.5% BSA, and model cells were treated with FFA (oleic acid:palmic acid = 2:1) (Sigma-Aldrich Co., St. Louis, MO, USA)dissolved in DMEM containing 0.5% BSA.

### 4.2. Measurement of Cell Viability

HepG2 cells were seeded at a density of 5 × 10^3^ cells/well in a 96-well plate. PCBG, PCB, and MPG (purity ≥98%; Figure 1) were isolated by the chemistry department of the Chinese Material Medicine Laboratory at the Capital Medical University (Beijing, China) and QCT was purchased from National Institutes for Food and Drug Control (purity = 97.3%; Figure 1D). They were dissolved in dimethyl sulfoxide (DMSO) (Sigma-Aldrich, St. Louis, MO, USA) and diluted to suitable concentrations in DMEM containing 0.5% BSA (DMSO < 0.1%). To determine the modeling concentration and the non-toxic concentration for the cells, FFA (0.6, 0.7, 0.8, and 1 mM), in addition to PCB, PCBG, and MPG (10, 50, 100, and 250 μM) were then added to each well. The plates were then incubated for 24 h at 37 °C under 5% CO_2_. Then, 10 μL of 3-(4,5-dimethylthiazol-2-yl)-2,5-diphenyltetrazolium bromide (MTT) solution (5 mg/mL) (Sigma-Aldrich Co., St. Louis, MO, USA) was added to each well and the cells were cultured for another 4 h. The supernatant was removed and 100 μL of DMSO/well was added to dissolve the intracellular crystalline formazan product. Cell viability was determined by measuring the absorbance at 490 nm using a SpectraMax Plus 384 Microplate Reader (Molecular Devices, Sunnyvale, CA, USA).

### 4.3. Oil Red O Staining

The fat accumulation in the HepG2 cells was determined by Oil Red O staining using a commercial kit (Nanjing Jiancheng Bioengineering Institute, Nanjing, China). The cells were treated for 24 h with 0.8 mM FFA and various concentrations of PCBG (0.1, 1, and 10 μM), PCB, and MPG (1, 10, and 100 μM). Cells were rinsed with cold phosphate-buffered saline (PBS) (Hyclone, South Logan, UT, USA), then stained with fresh Oil Red O working solution for 20 min. Stained cells were washed with PBS prior to microscopic observation using a Nikon 80i upright microscope (Nikon, Tokyo, Japan). To quantify, 250 μL of DMSO was added to the dried plates, and the optical density was measured at 510 nm.

### 4.4. Biochemical Assay

HepG2 cells were seeded into six-well plates at 2 × 10^5^ cells per well. After 24 h of incubation, the culture medium was removed and treated with 0.8 mM FFA and various concentrations of PCBG (0.1, 1, and 10 μM), PCB, and MPG (1, 10, and 100 μM). Cells were collected and made into a homogenate with PBS. The levels of TC, TG, ALT, AST, MDA, SOD, and GSH-Px were determined with commercial kits (Nanjing Jiancheng Bioengineering Institute, Nanjing, China) and normalized by protein content (mg/mL).

### 4.5. Western Blots Analysis

To detect the proteins of cells, cells (2 × 10^6^) were plated into 100-mm dishes and incubated overnight, before being starved for 12 h in DMEM with 0.5% BSA and submitted to different treatments as described above. After each 24-h treatment, cells were collected and washed with PBS and lysed on ice in Radio-Immunoprecipitation Assay (RIPA) buffer with a protease and phosphatase inhibitor cocktail for 15 min. Cell lysates were centrifuged at 12,000 rpm for 10 min, before the supernatant was collected and the protein content of each lysate was measured using a bicinchoninic (BCA) protein assay kit (Beijing Biosynthesis Biotechnology Co., Ltd., Beijing, China). Proteins (20 μg/lane) were subjected to SDS-PAGE with 10% resolving gel. The separated proteins on gels were then transferred onto a polyvinylidene difluoride (PVDF) membrane (Merch/Millipore, Schwalbach, Germany). After blocking nonspecific binding with 5% fat-free milk or 2% BSA solution, the membranes were incubated with antibodies against SIRT1 (sc-74465), AMPK (sc-25792), p-AMPK (sc-33524), SREBP1c (sc-365513), PPARα (sc-130640), and glyceraldehyde 3-phosphate dehydrogenase (GAPDH; sc-32233) (Santa Cruz, CA, USA) at 4 °C overnight. After being washed with Tris-buffered saline/Tween (TBST) four times, the membranes were incubated with horseradish peroxidase (HRP)-conjugated goat anti-rabbit immunoglobulin G (IgG) or rabbit anti-mouse IgG (Zhongshan Goldenbridge, Beijing, China) for 1 h at room temperature. The blots were incubated in Immobilon Western Chemiluminescent HRP Substrate and exposed to an X-film to form an image. The protein bands were quantitated using the Image J software. (Version 1.51k, National Institutes of Health, Bethesda, MD, USA)

### 4.6. Quantitative Real-Time Polymerase Chain Reaction (qRT-PCR)

HepG2 cells were plated at a density of 3 × 10^5^ cells/well in a six-well plate and incubated overnight, before being starved for 12 h in DMEM with 0.5% BSA and used for different treatments as described above; one group was treated with QCT (10 μM) as a comparison. Total RNA from HepG2 cells was extracted using an RNAprep pure Cell/Bacteria Kit (Tiangen Biotech Co., Beijing, China). The purity and concentration of RNA were determined with a Genova Nano spectrophotometer (BIBBY JENWAY, Staffordshire, UK). Complementary DNA (cDNA) was synthesized with a FastQuant RT Kit (With gDNase) (Tiangen Biotech Co., Beijing, China) according to the manufacturer’s protocols. The relative levels of mRNA to *GAPDH* were analyzed using an SYBR fast universal qPCR kit (KAPA Biosystems, MA, USA) and specific primers. The primer sequences are shown in Table 2. The qRT-PCR was performed on an ABI Quant 5 PCR system using the 2^−^^ΔΔCt^ method. *GAPDH* was used as the normalized reference gene.

### 4.7. Molecular Docking

The docking studies of PCB, PCBG, MPG, and reference compound, QCT, were performed with energy-metabolism-related molecular targets, including SIRT1 (Protein Data Bank identifier (PDB ID): 4ZZJ), AMPK (PDB ID: 4ZHX), PPARα (PDB ID: 3KDU), FAS (PDB ID: 5C37), ACC1 (PDB ID: 3TVU), and SCD1 (PDB ID: 4ZYO). The crystal structures were obtained from the Research Collaboratory for Structural Bioinformatics Protein Data Bank (RCSB PDB; http://www.rcsb.org), and were protonated and energy minimized using the AMBER FF99 force field [56]. The structures of compounds were drawn using ChemDraw Ultra 7.0 (CambridgeSoft, Perkin Elmer Inc., Waltham, MA, USA) and converted to three-dimensional (3D) structures with all proton and tripos force charges added to optimize the minimum energy conformation using SYBYL-X 1.2. The optimized conformation was used for the analysis of docking events with Surflex-Dock, which is a well-recognized method in the field of molecular docking. In this way, the virtual screening and ligand–receptor interaction were evaluated.

### 4.8. Statistics

Data were shown as mean ± standard deviation of at least three independent experiments. One-way ANOVA and a Student’s *t*-test were used to evaluate statistical significance with the SPSS statistics 23.0 software. Values of *p* < 0.05 were considered as statistically significant.

## Figures and Tables

**Table 1:** Docking Results of Pinocembrin (PCB), Pinocembrin-7-*O-β*-d-Glucoside (PCBG), 5-Methoxy-Pinocembrin-7-*O-β-*d-Glucoside (MPG), and Quercetin (QCT).

Targets	Ligand	Docking Score	H-Bonds	Residue of Hydrogen Bond	Targets	Ligand	Docking Score	H-Bonds	Residue of Hydrogen Bond
SIRT1 (4ZZJ)	QCT	7.4850	6	GLY263, ASN465, GLU467, ARG276, GLU656	AMPK (4ZHX)	QCT	5.1547	6	GLU94, MET93, VAL96, LEU22, GLU100
	PCB	5.0529	3	ALA262, SER442, GLN294		PCB	3.7372	3	SER97, TYR95, ASP103
	PCBG	7.2696	9	ARG466, GLU467, ASN465, GLY263, ALA262, GLN345, GLN294		PCBG	5.6765	7	VAL96, TYR95, SER97, ASP103, GLU100
	MPG	7.0478	4	SER442, ASP272, ARG274		MPG	6.3571	2	GLU100, ASN144
PPARα (3KDU)	QCT	5.7407	1	THR279	FAS (5C37)	QCT	5.3856	3	ARG1917, ASN1945
	PCB	3.1022	3	ALA333, THR279, CYS275		PCB	4.4753	1	ILE1946
	PCBG	4.8008	4	TYR464, PHE273, PHE351, MET355		PCBG	7.2480	4	ARG1917, VAL1973, GLY1895, PHE1896
	MPG	6.5830	5	TYR464, MET355, CYS276, GLU269, PHE273		MPG	8.1074	5	ARG1917, VAL1973, PHE1896, GLY1895, GLY1897
ACC1 (3TVU)	QCT	4.6247	8	SER1999, ARG1954, ARG1996, PHE1956, GLY1958, MET1963	SCD1 (4ZYO)	QCT	5.5432	3	LYS189, ASP156
	PCB	3.3302	4	ARG1996, GLY1998, SER1999, PHE1956		PCB	5.5185	2	LYS189, ARG155
	PCBG	4.8381	6	PHE1956, SER1999, ARG1996, ARG1954, GLU2026		PCBG	5.8544	5	LYS194, ASP156, LYS189, ASN75, ASN148
	MPG	4.5763	2	PHE1956, ARG1954		MPG	4.9069	4	LYS194, LYS189, GLU152

SIRT1—silent mating type information regulation 2 homolog 1; PPARα—peroxisome proliferator-activated receptor α; ACC1—acetyl-CoA carboxylase; AMPK—AMP-activated protein kinase; FAS—fatty acid synthase; SCD1—stearoyl-CoA desaturase 1.

**Table 2:** The Primers Used for qRT-PCR.

Gene Name	Forward Primer (5′–3′)	Reverse Primer (5′–3′)	Reference
*SIRT1*	GCCAGAGTCCAAGTTTAGAAGA	CCATCAGTCCCAAATCCAG	[52]
*AMPK*	CAGGCATATGGTGGTCCATAGAG	TCATGGGATCCACCTGCAGC	[18]
*SREBP1c*	ATACCACCAGCGTCTACC	CACCAACAGCCCATTGAG	[48]
*PPARα*	AGCAAGGAAGGGTTGTGGCAAA	ATGGACTCGGAAGCAGGAAGGT	[53]
*FAS*	CGGCTCGCCCACCT	CGGGCCGCAAAGC	[48]
*ACC*	GCTGCTCGGATCACTAGTGAA	TTCTGCTATCAGTCTGTCCAG	[54]
*SCD1*	CCTCTACTTGGAAGACGACATTCGC	GCAGCCGAGCTTTGTAAGAGCGGT	[54]
*GAPDH*	TGCACCACCAACTGCTTAGC	GGCATGGACTGTGGTCATGAG	[55]

## Supplementary Materials

The following are available online at http://www.mdpi.com/1422-0067/19/9/2555/s1, Figure S1: Molecular interactions of PCB, PCBG, MPG, and QCT binding with SIRT1, AMPK, PPARα, FAS, ACC1 and SCD1.
