# Supplementary material for: Flavonones from Penthorum chinense Ameliorate Hepatic Steatosis by Activating the SIRT1/AMPK Pathway in HepG2 Cells
Source: Int J Mol Sci. 2018 Aug 28;19(9):2555. doi: 10.3390/ijms19092555 (PMC6165420; doi:10.3390/ijms19092555)
Supplement: Supplementary file 1 [file ijms-19-02555-s001.pdf]

Supplementary Materials

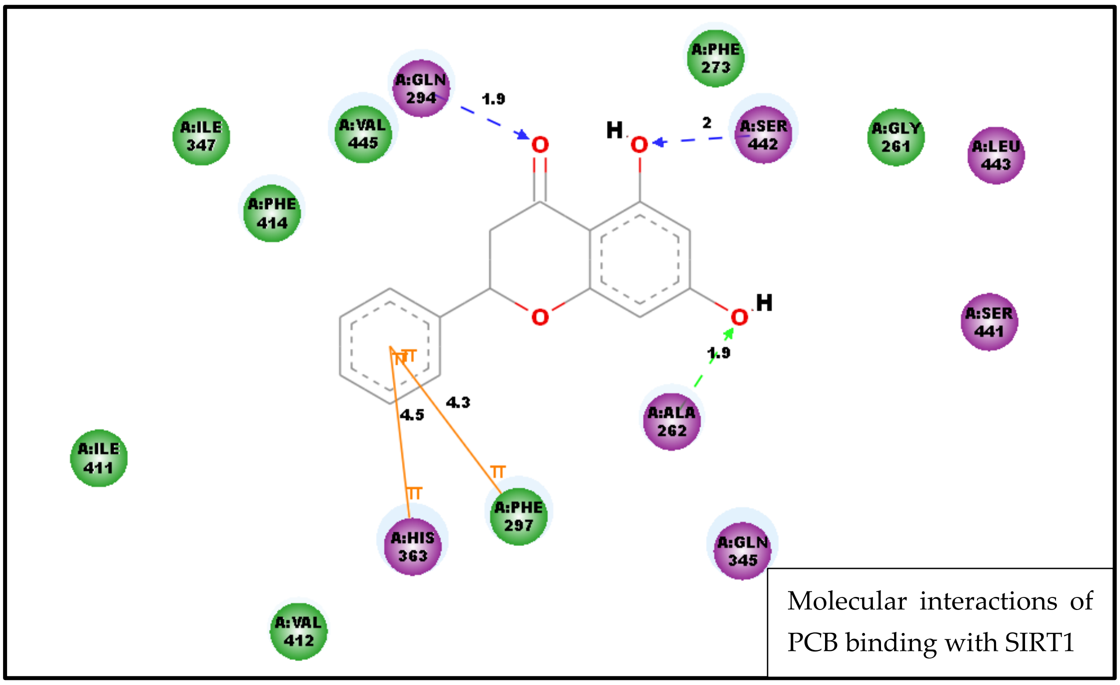

Molecular interactions of PCB binding with SIRT1

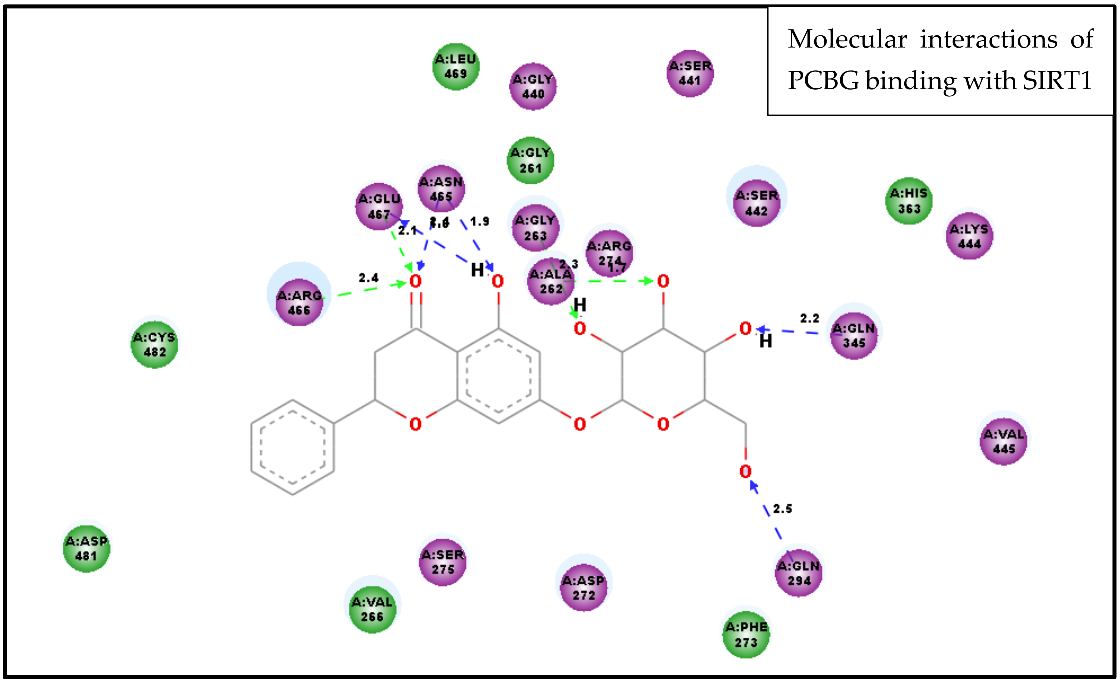

Molecular interactions of PCBG binding with SIRT1

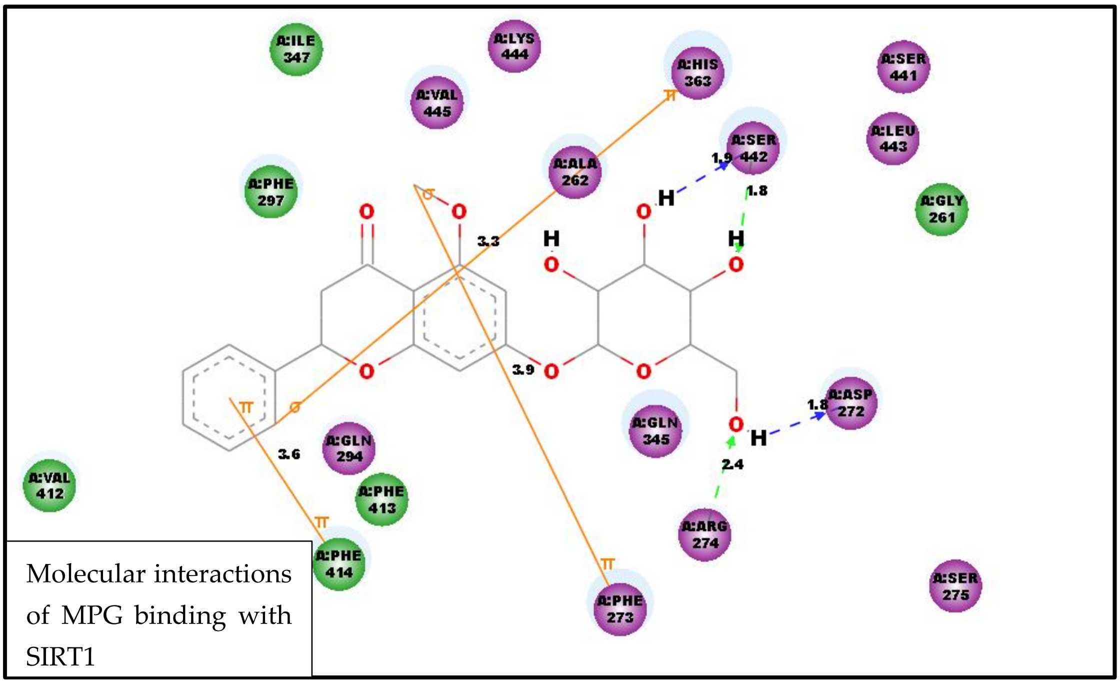

Molecular interactions of MPG binding with SIRT1

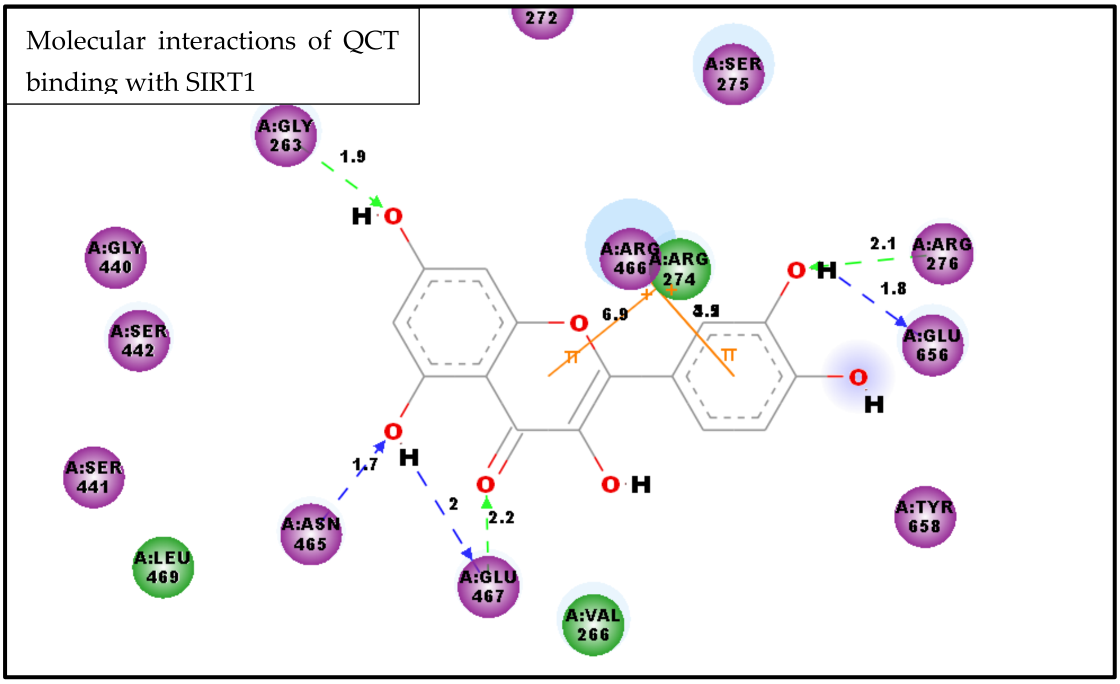

Molecular interactions of QCT binding with SIRT1

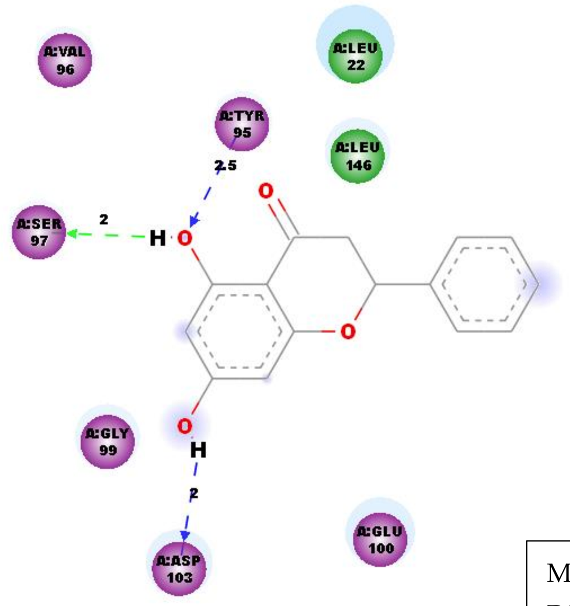

Molecular interactions of PCB binding with AMPK

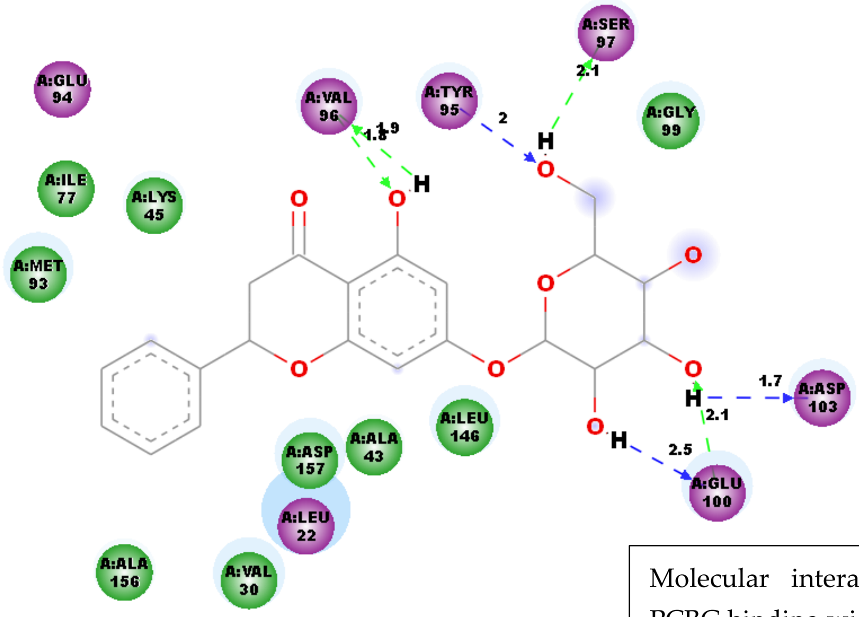

Molecular interactions of PCBG binding with AMPK

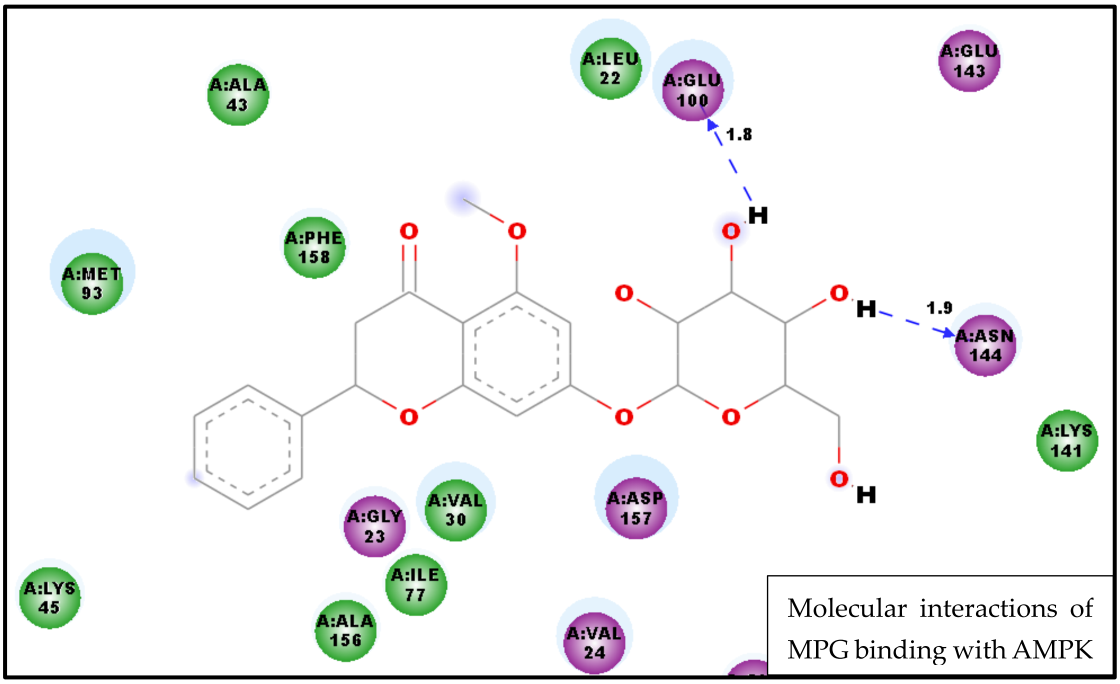

Molecular interactions of MPG binding with AMPK

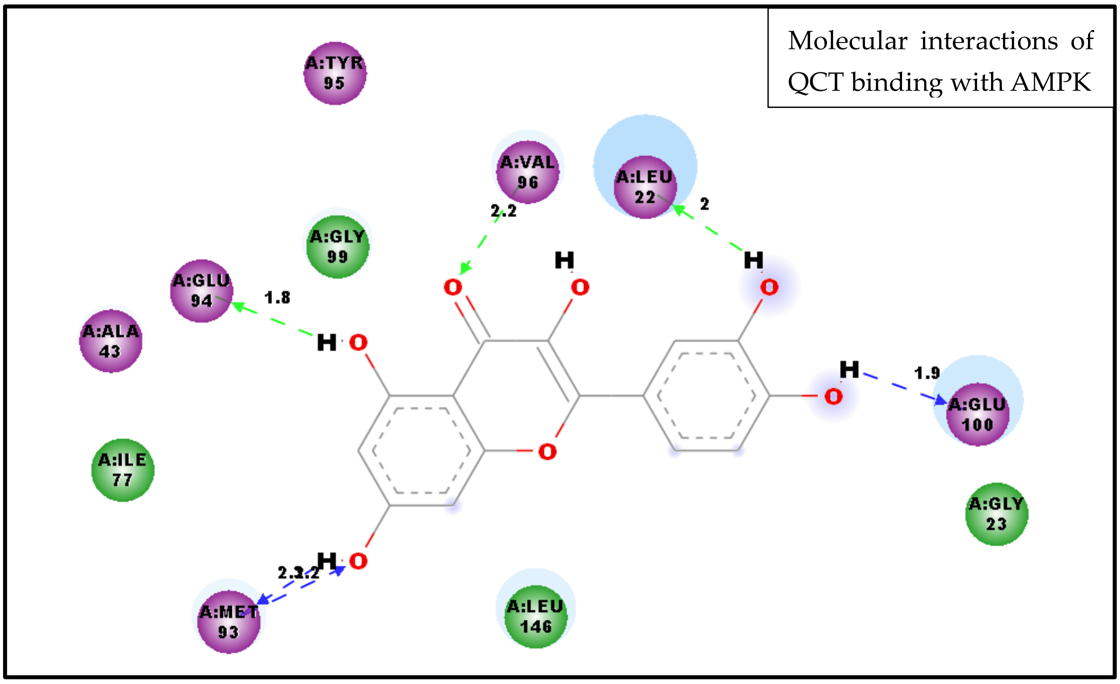

Molecular interactions of QCT binding with AMPK

Molecular interactions of PCB binding with PPARα

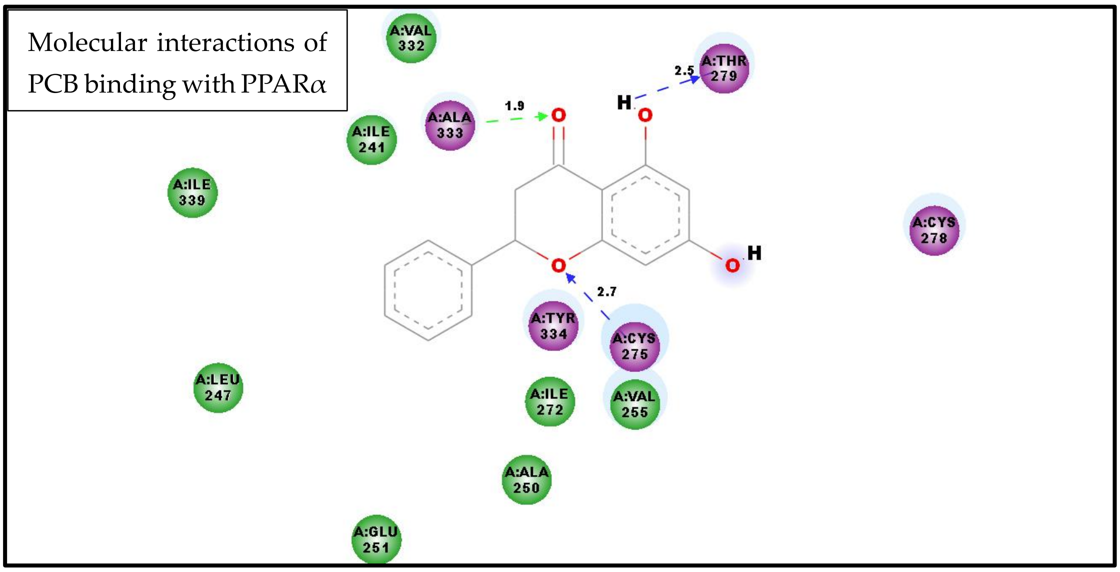

Molecular interactions of PCBG binding with PPARα

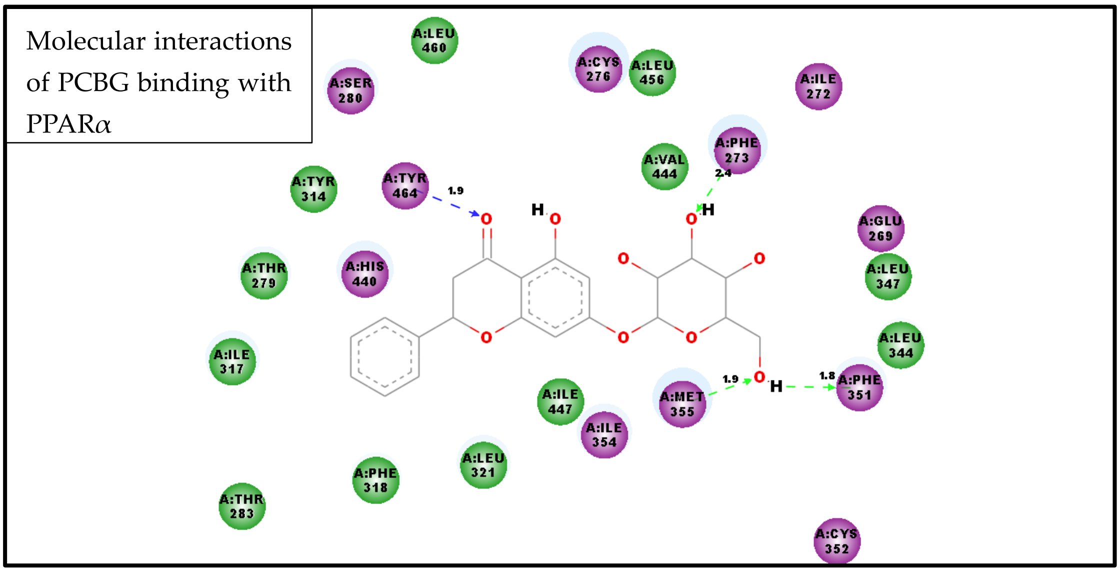

Molecular interactions of MPG binding with PPARα

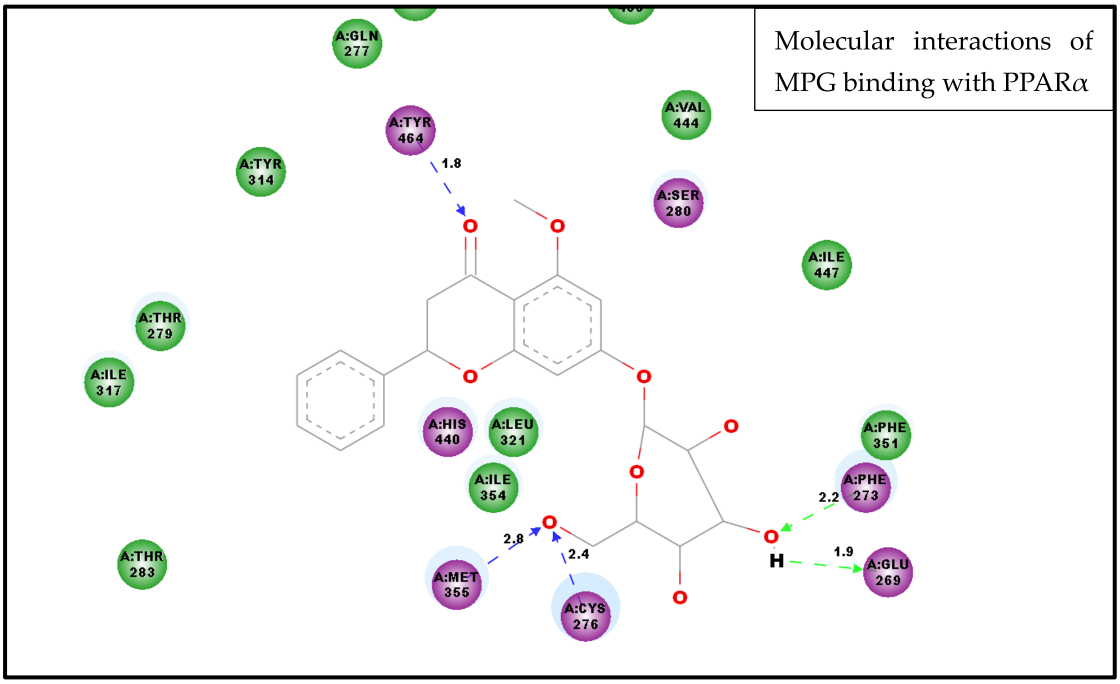

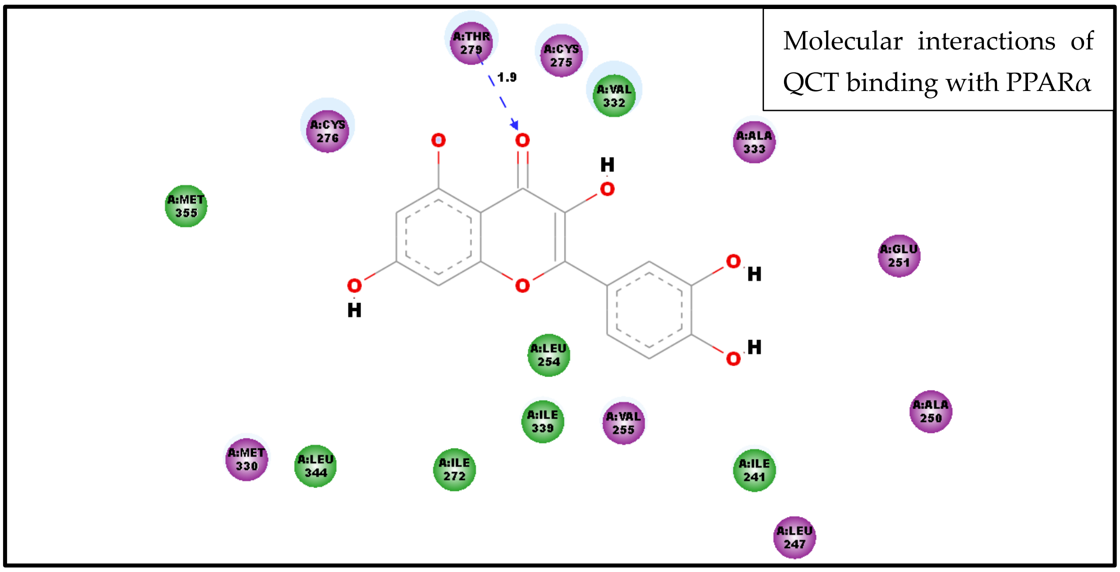

Molecular interactions of QCT binding with PPARα

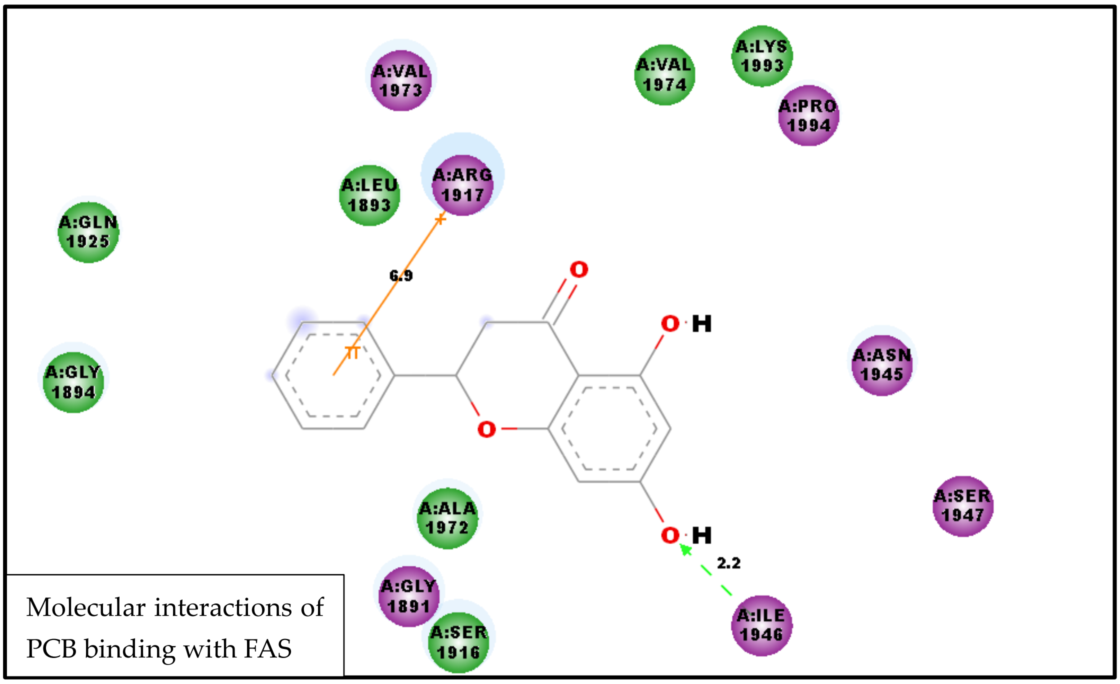

Molecular interactions of PCB binding with FAS

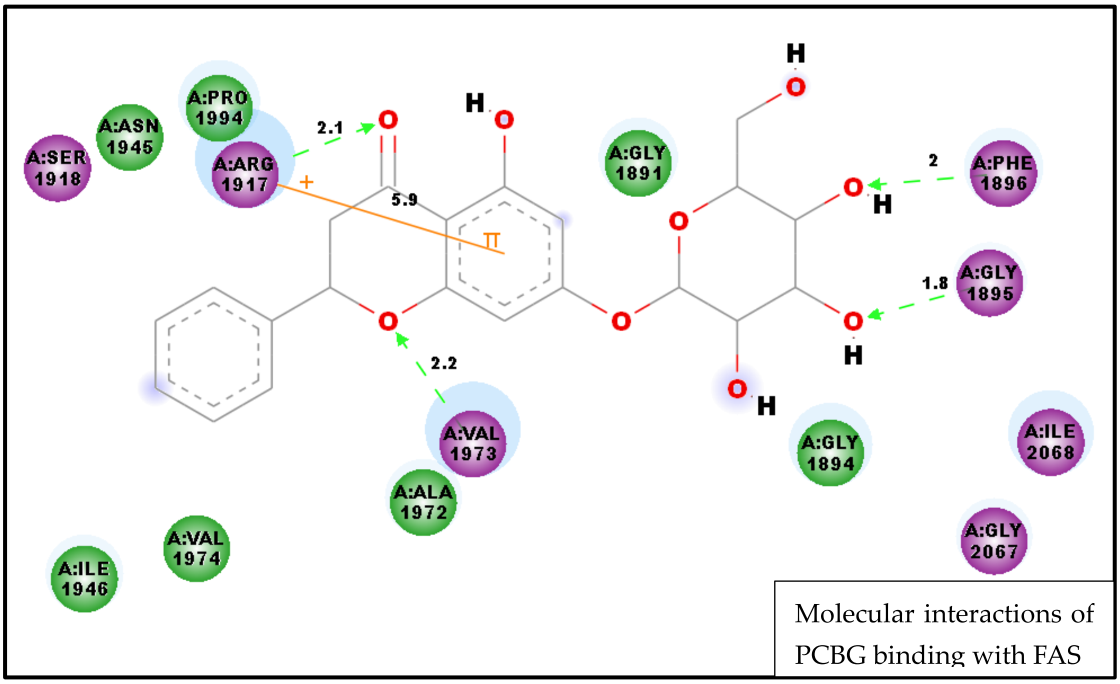

Molecular interactions of PCBG binding with FAS

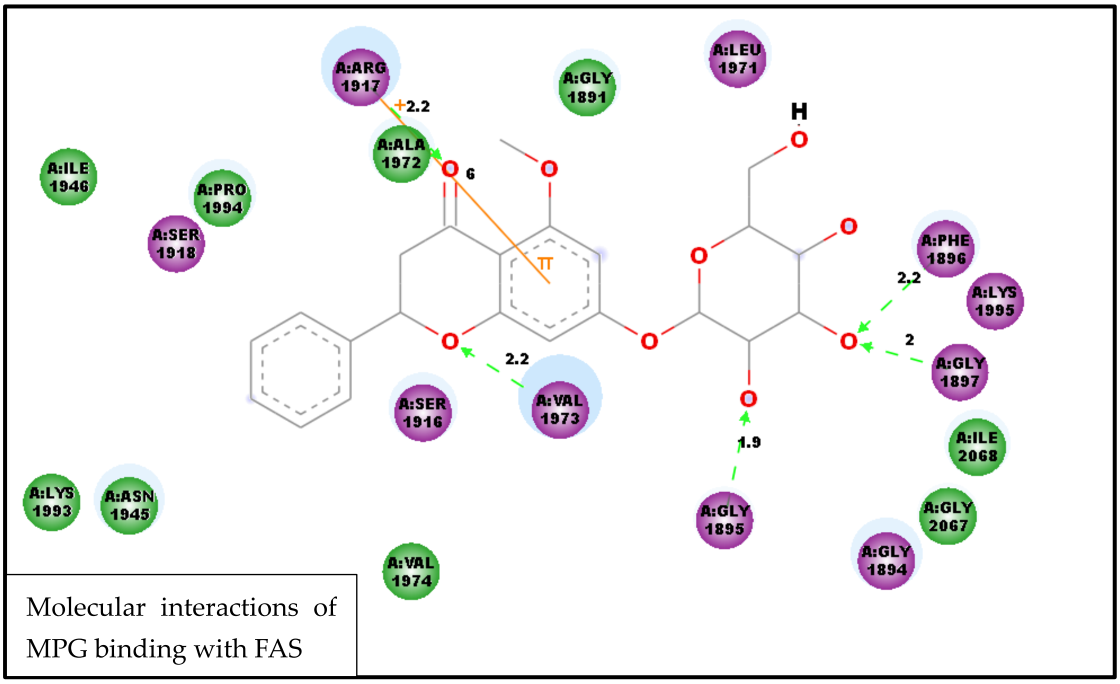

Molecular interactions of MPG binding with FAS

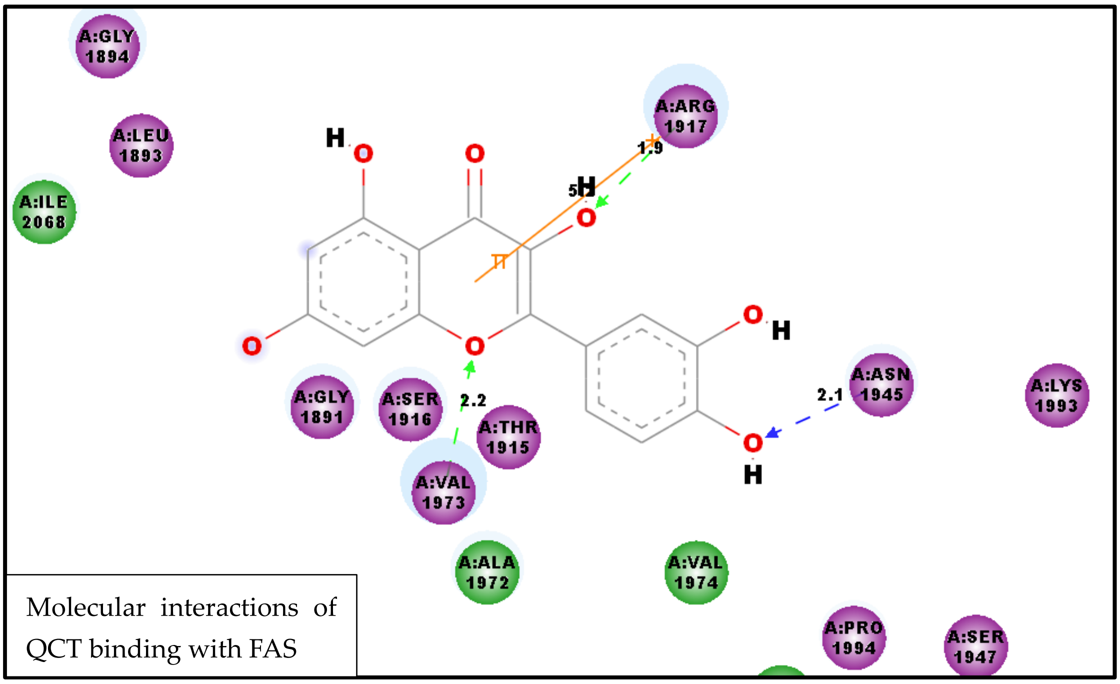

Molecular interactions of QCT binding with FAS

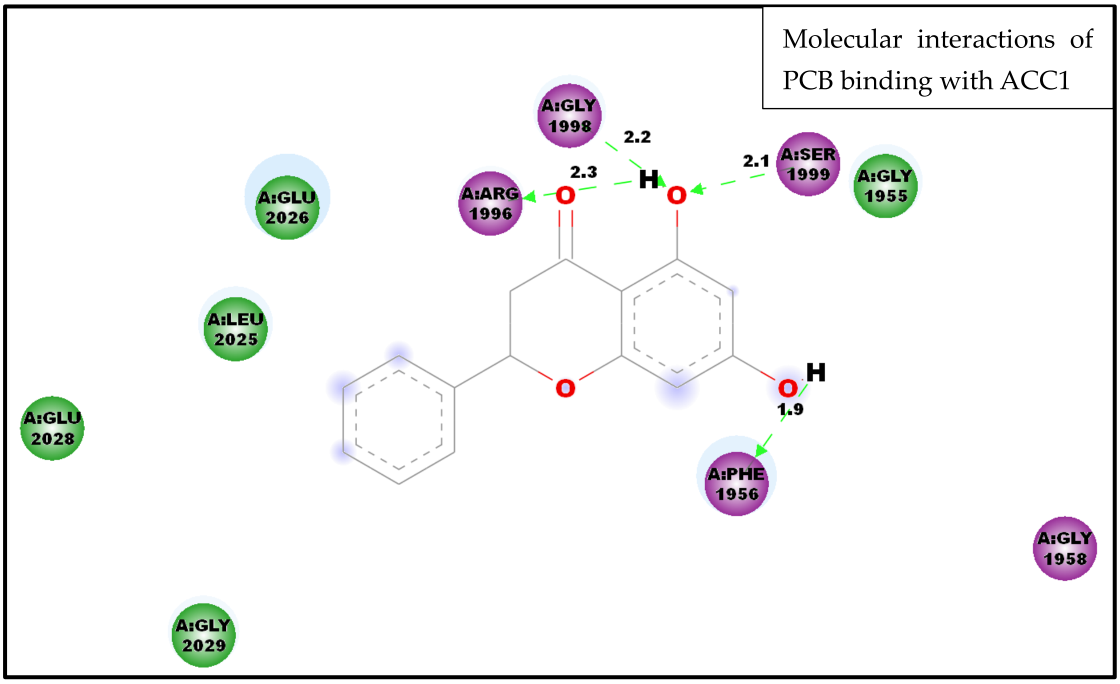

Molecular interactions of PCB binding with ACC1

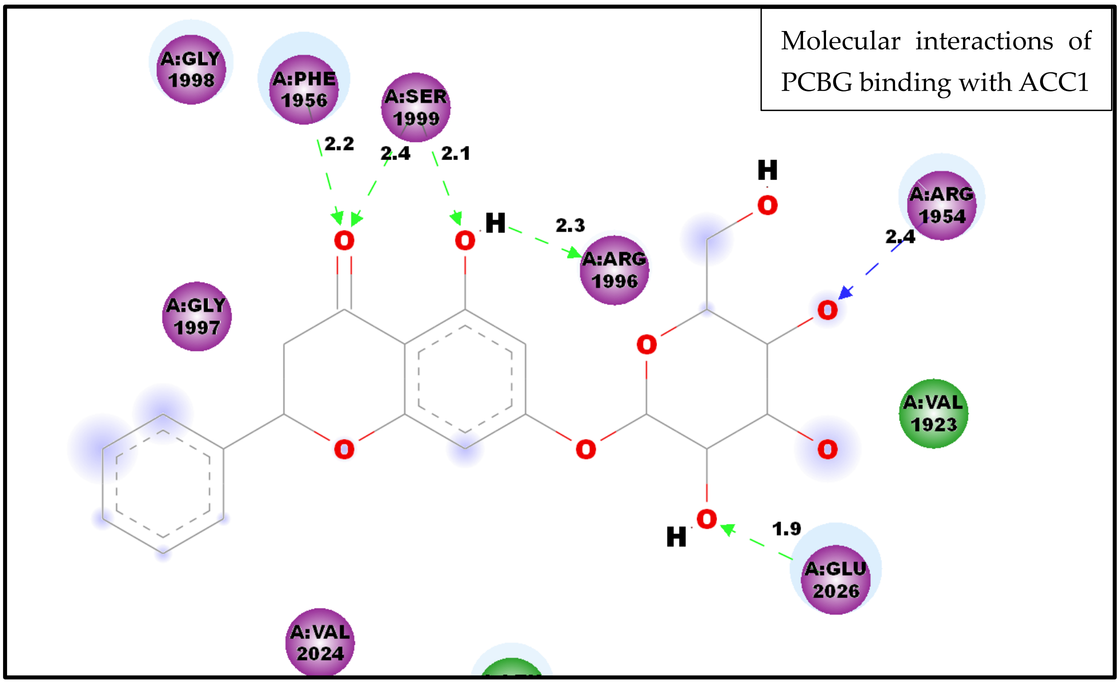

Molecular interactions of PCBG binding with ACC1

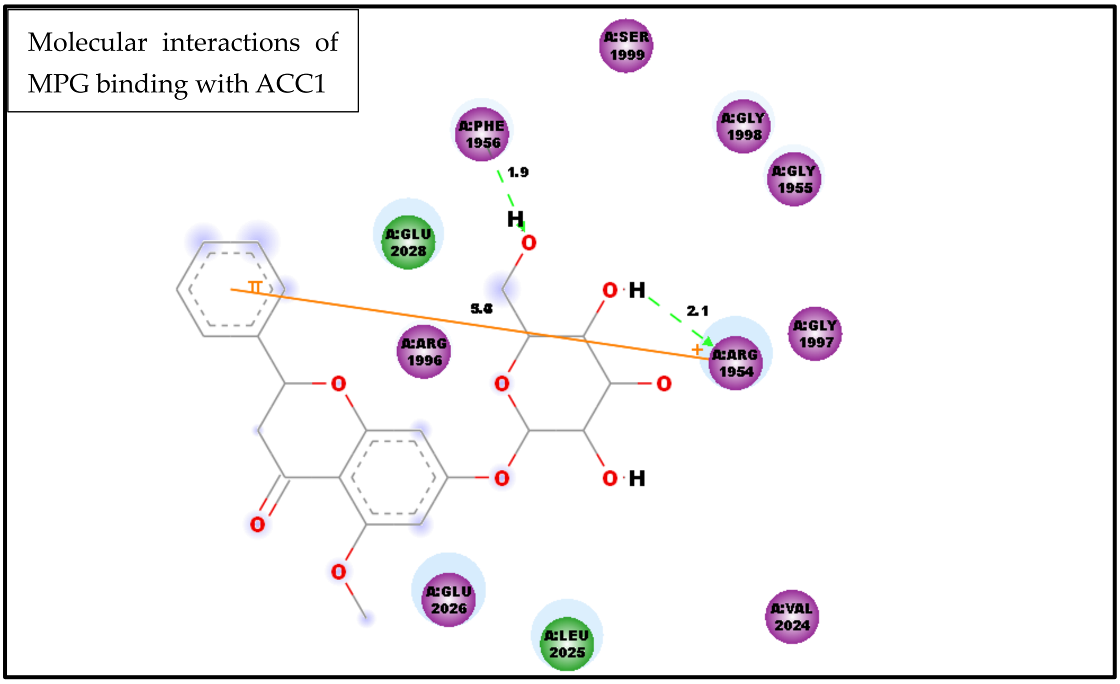

Molecular interactions of MPG binding with ACC1

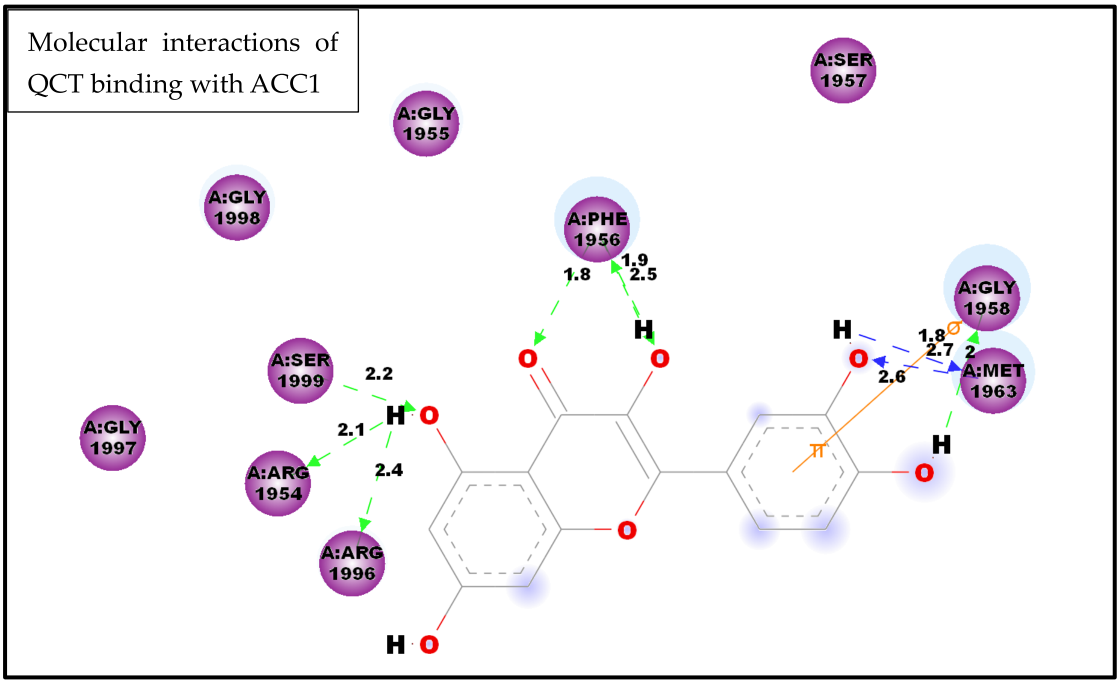

Molecular interactions of QCT binding with ACC1

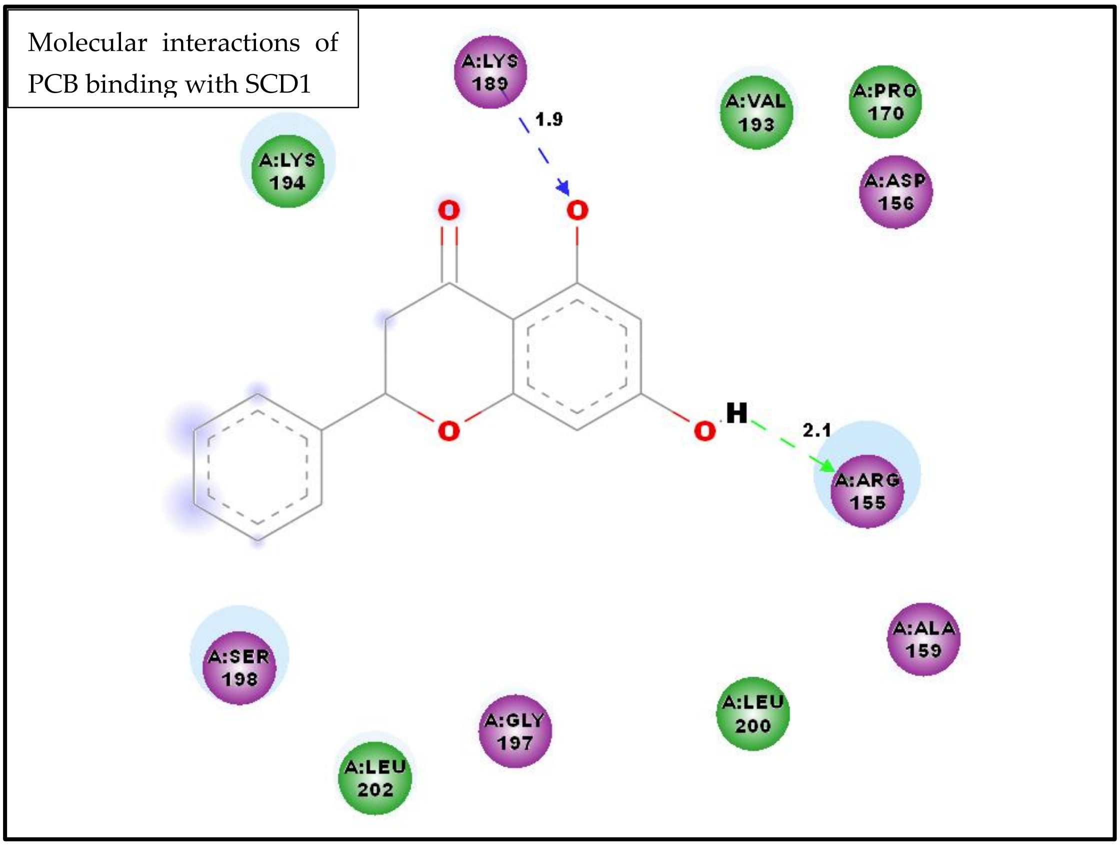

Molecular interactions of PCB binding with SCD1

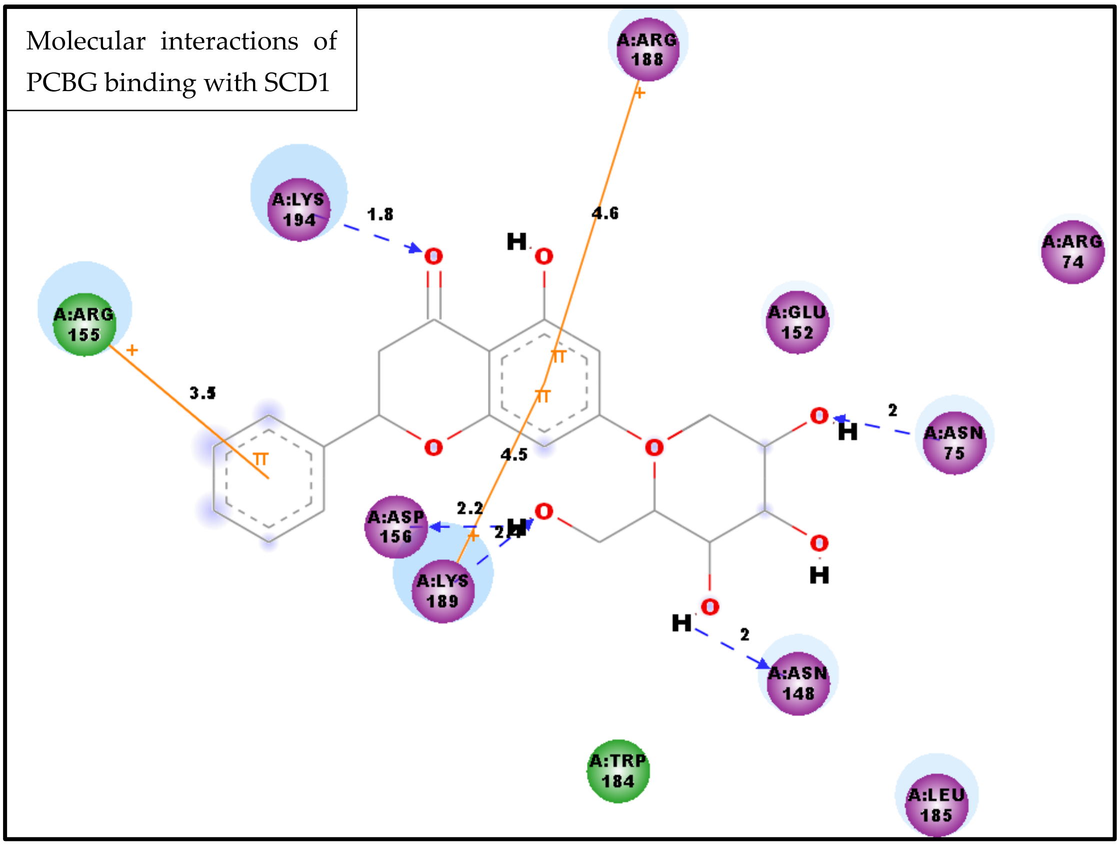

Molecular interactions of PCBG binding with SCD1

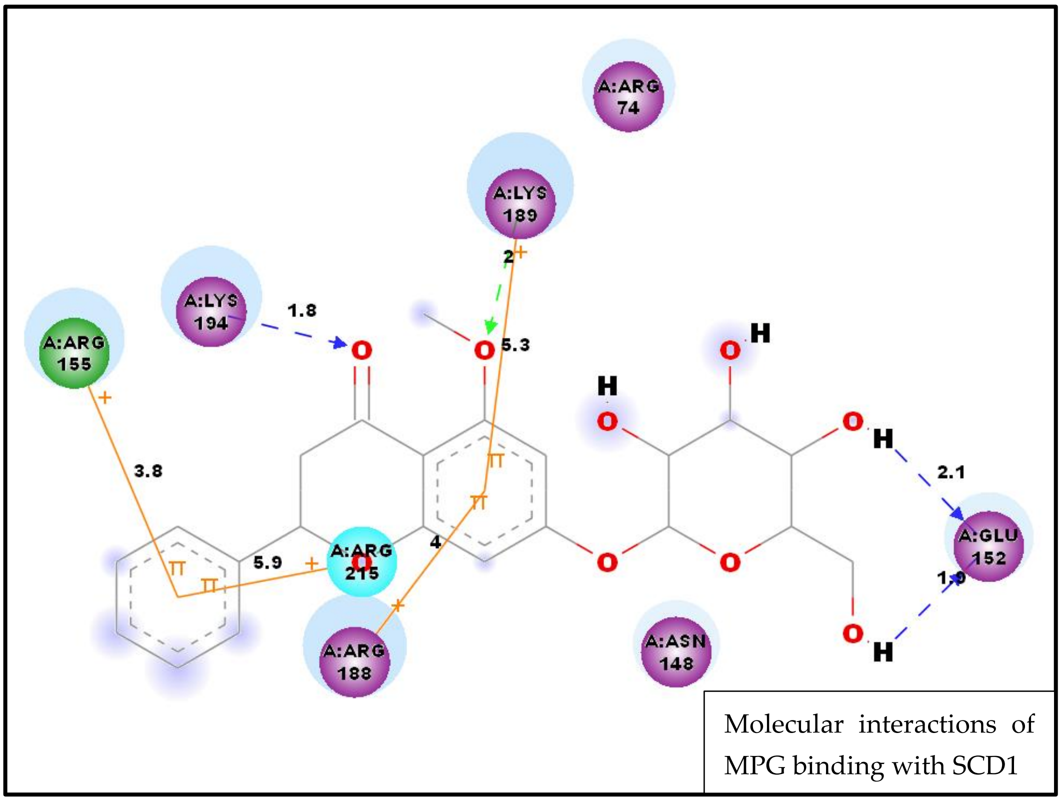

Molecular interactions of MPG binding with SCD1

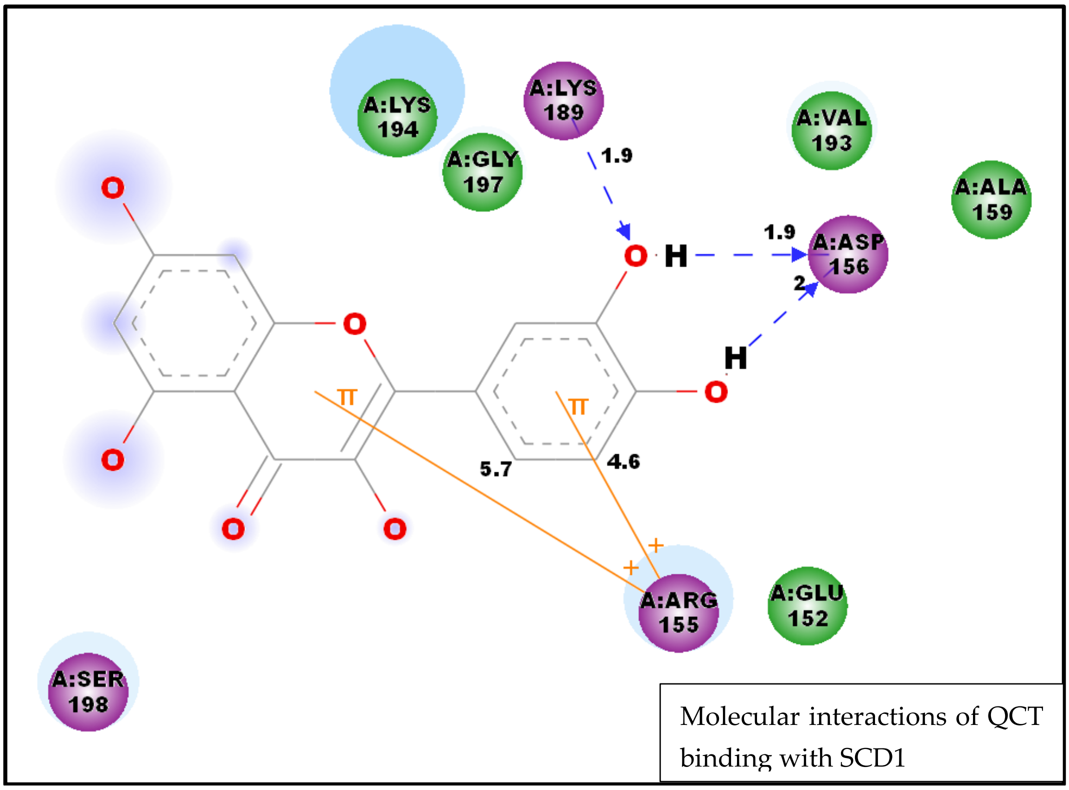

Figure S1. Molecular interactions of PCB, PCBG, MPG, and QCT binding with SIRT1, AMPK, PPARα, FAS, ACC1 and SCD1.
